# Supplementary material for: The interactome of intact mitochondria by cross-linking mass spectrometry provides evidence for coexisting respiratory supercomplexes
Source: Mol Cell Proteomics. 2017 Dec 8;17(2):216–32. doi: 10.1074/mcp.RA117.000470 (PMC5795388; doi:10.1074/mcp.RA117.000470)
Supplement: Supplemental Data [file supp_RA117.000470_133922_0_supp_23978_fzffwf.zip › spectra_annotation/mito_DR_spectra_annotation/360-1-7-1-3-1.pdf]

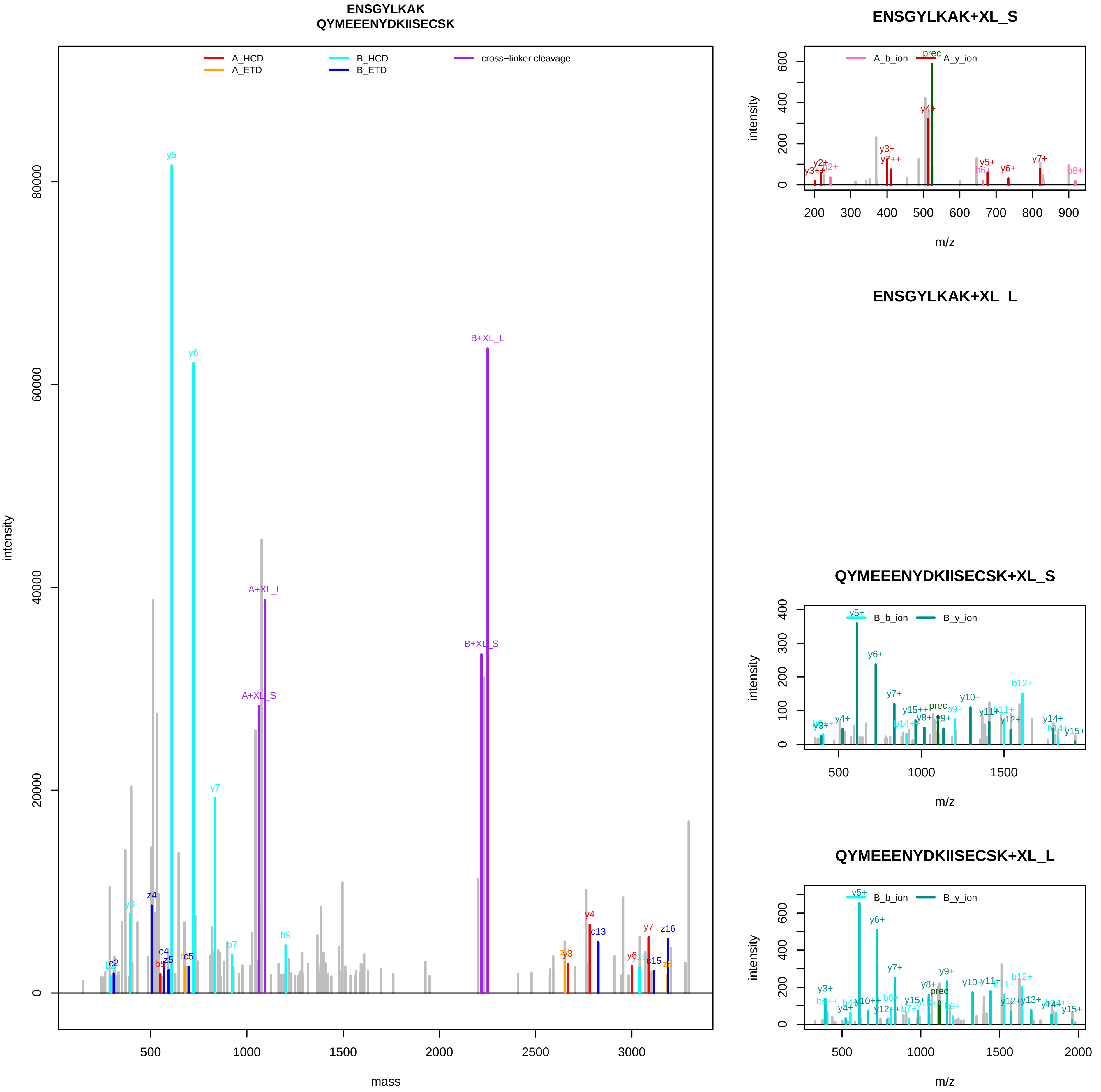

ENSGYLKAK
QYMEEENYDKIISECSK
A_HCD
A_ETD
B_HCD
B_ETD
cross−linker cleavage
intensity
mass
0
20000
40000
60000
80000
500
1000
1500
2000
2500
3000
y5
y6
B+XL_L
B+XL_S
A+XL_L
A+XL_S
y7
y3
z4
c4
z5
c5
c2
b7
b9
y4
c13
y7
z16
y6
c15
y8
ENSGYLKAK+XL_S
A_b_ion
A_y_ion
prec
y4+
y3+
y7++
y2+
b2+
y3++
y5+
b6+
y6+
y7+
b8+
m/z
ENSGYLKAK+XL_L
QYMEEENYDKIISECSK+XL_S
B_b_ion
B_y_ion
y5+
y6+
y7+
y10+
b12+
y15++
y14+
y15+
QYMEEENYDKIISECSK+XL_L
